# Bioactivities and Mechanism of Actions of *Dendrobium officinale*: A Comprehensive Review

**DOI:** 10.1155/2022/6293355

**Published:** 2022-09-16

**Authors:** Xiaoyu Xu, Cheng Zhang, Ning Wang, Yu Xu, Guoyi Tang, Lin Xu, Yibin Feng

**Affiliations:** ^1^School of Chinese Medicine, Li Ka Shing Faculty of Medicine, The University of Hong Kong, Hong Kong 999077, China; ^2^School of Pharmacy, Shanghai University of Traditional Chinese Medicine, Shanghai 201203, China; ^3^Engineering Research Center of Shanghai Colleges for TCM New Drug Discovery, Shanghai 201203, China

## Abstract

*Dendrobium officinale* has a long history of being consumed as a functional food and medicinal herb for preventing and managing diseases. The phytochemical studies revealed that *Dendrobium officinale* contained abundant bioactive compounds, such as bibenzyls, polysaccharides, flavonoids, and alkaloids. The experimental studies showed that *Dendrobium officinale* and its bioactive compounds exerted multiple biological properties like antioxidant, anti-inflammatory, and immune-regulatory activities and showed various health benefits like anticancer, antidiabetes, cardiovascular protective, gastrointestinal modulatory, hepatoprotective, lung protective, and neuroprotective effects. In this review, we summarize the phytochemical studies, bioactivities, and the mechanism of actions of *Dendrobium officinale*, and the safety and current challenges are also discussed, which might provide new perspectives for its development of drug and functional food as well as clinical applications.

## 1. Introduction

*Dendrobium officinale* Kimura et Migo, belonging to the *Dendrobium* of *Orchidaceae* genus, is widely used as a medicinal and functional food product [1]. It originated from Nanling Mountains and Yungui Plateau in China, and its cultivation migrated northward or eastward subsequently [2]. *Dendrobium officinale* was originally used as a tonic herbal medicine to treat stomach disorders and promote the secretion of body fluid in Chinese medicine [1]. It also has a long history as a food ingredient in Yunnan and Zhejiang Province in China, and the main ways of consumption are making instant food, soup, dishes, juices, tea, and wine. In particular, the dried stem of *Dendrobium officinale* (*Dendrobii officinalis*) has been documented in Chinese Pharmacopoeia for medicinal usage and is officially listed in “Medicine and Drug Homology,” which indicates that *Dendrobium officinale* might be feasible for long-term consumption with high safety [3].

Increasing pharmacological studies have found that *Dendrobium officinale* has a high nutritional and medicinal value, such as antioxidant, immune-regulatory, anti-inflammatory, anticancer, antidiabetic, and hepatoprotective activities [4, 5]. These health benefits are mainly attributed to its abundant bioactive compounds, such as flavonoids, bibenzyls, polysaccharides, and alkaloids [6]. As a natural plant product, *Dendrobium officinale* poses little toxicity and side effects to human health, and it could combine with other herbal medicines in Chinese medicine decoction for the treatment of diseases. Since the present chemical drugs and therapy could cause some side effects in patients, it is essential to develop natural-derived drugs and adjuvant supplements with fewer side effects for patients. Hence, phytochemicals and herbal therapeutics have gained lots of attention for investigation in various disease treatments. *Dendrobium officinale* might be a promising dietary supplement and functional food in the prevention and management of diseases, and its bioactivities and mechanisms of action are worthy of exploration [7]. This review summarized the updated knowledge of the phytochemical studies, bioactivities, health benefits, related mechanism of action, and safety of *Dendrobium officinale*. The current challenge and outlooks of *Dendrobium officinale*-related research are also discussed, providing new and critical viewpoints for developing medicinal and functional food in the future.

## 2. Phytochemical Studies

### 2.1. Bioactive Compounds

**Figure 1:**
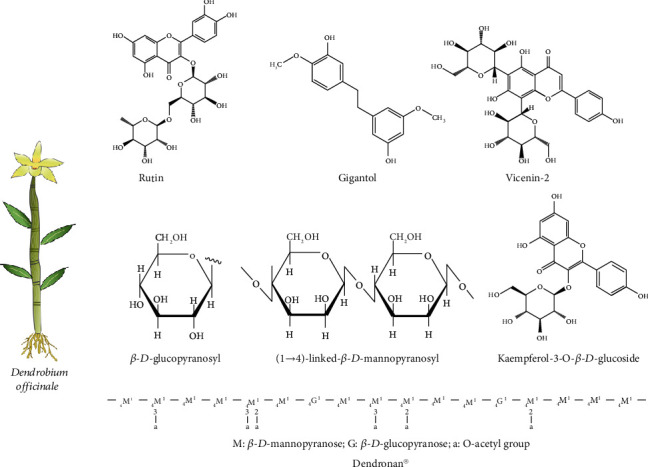
The chemical structures of several compounds found in different parts of *Dendrobium officinale.*

A large body of studies shows that *Dendrobium officinale* contains various bioactive compounds, such as polysaccharides, flavonoids, bibenzyls, and alkaloids [4, 7–9] (Figure 1). The tissue analysis found that the stems, leaves, and protocorm-like bodies of *Dendrobium officinale* had the highest content of polysaccharides, flavonoids, and alkaloids, respectively [10]. Among the bioactive compounds, polysaccharides are the major medicinal compound that is often utilized to investigate the therapeutic effects of *Dendrobium officinale.* It is mainly isolated from the stems of *Dendrobium officinale* with a yield rate of over 30% [11]. The chemical analysis showed that the polysaccharides mainly contained mannose and glucose with a structure of (1⟶4)-linked-*β*-*D*-mannopyranosyl and *β*-D-glucopyranosyl residues [12, 13]. Dendronan® is a new polysaccharide *O*-acetyl-glucomannan isolated from *Dendrobium officinale* with a relatively detailed chemical structure, and it was identified as the ratio of mannose to glucose (6.9 : 1) [14]. However, some polysaccharides with large molecular weight or absence of certain chemical groups might have low biological activities, and thus, some modifications could be considered for improving the bioavailability of polysaccharides from *Dendrobium officinale*, such as fermentation, degradation, or grafting [15, 16]. Furthermore, the relationship between structural characteristics and biological properties of *Dendrobium officinale* polysaccharides needs more in-depth investigation.

Additionally, the metabolic profile of *Dendrobium officinale* found that leaves contained more flavonoids than other parts, and flavonoids were considered the important antioxidant source [17–19]. A total of 14 major phenolic compounds including 1 quercetin-type flavonol, rutin, and 13 apigenin-type flavones like apigenin 6-*C*-*β*-*D*-glucoside-8-*C*-*α*-L-rhamnoside were identified from the leaves of *Dendrobium officinale*. And the major flavonoid compound was rutin with a content of 1.33 to 2.89 mg/g from leaves [19]. Moreover, naringenin was the flavonoid compound only found produced in the stems of *Dendrobium officinale* [10].

Bibenzyl is one of the most active ingredients in *Dendrobium officinale*, and the gigantol and dendrocandin are the most common bibenzyl compounds from *Dendrobium officinale* [4]. The phytochemical study found that the root tissues of *Dendrobium officinale* contained the highest amount of bibenzyl, such as erianin and gigantol. And the transcriptomic analysis revealed that cytochrome P450 genes and other enzymatic genes were functionally associated with the biosynthesis and accumulation of bibenzyl, which might help increase the content of bibenzyl for drug production and industrialization of *Dendrobium officinale* [20]. Several bibenzyl compounds have also been found in the leaves of *Dendrobium officinale*, such as the new derivate denofficin, dendrocandin B, 4,4′-dihydroxy-3,5-dimethoxy bibenzyl, gigantol, and densiflorol [21].

Alkaloids were found abundant in protocorm-like bodies of *Dendrobium officinale*, which might be more available for producing alkaloids than other organs. The study also found that the enzymes involved in the alkaloid biosynthesis were strictosidine *β*-*D*-glucosidase, geissoschizine synthase, and vinorine synthase in *Dendrobium officinale* [10]. Additionally, the key enzyme-encoding genes associated with the alkaloid biosynthesis had higher activities in the leaves than that in the stems of *Dendrobium officinale* [22].

Due to the increasing demand and rare resources of wild type, there are more and more adulterations of *Dendrobium officinale*, and it negatively affects the sustainable utilization of this medicinal plant and food resource and increases the potential health risk of using cheaper and poorer products. The composition of ingredients contributes to the quantitative chemotypic variation and characteristics within difference [23]. Hence, some methods targeting the specific compounds of *Dendrobium officinale* have been developed for distinguishment. For example, the quantifications of naringenin, bibenzyls, and the ratios of mannose to glucose of polysaccharides could be used as key elements to distinguish from other similar spices [24]. Additionally, the combined analysis of HPLC fingerprints, HPLC-ESI-MS, and HPTLC found that violanthin and isoviolanthin were specific components for *Dendrobium officinale*, which could distinguish it from *Dendrobium devonianum* [25].

Overall, the phytochemical studies found abundant bioactive components in *Dendrobium officinale*, and they are closely associated with various bioactivities and health benefits. The identification of chemical structures of some major compounds as well as biosynthesis-related gene encoding enzymes is important for the exploration and protection of *Dendrobium officinale.* On the other hand, the chemical composition of *Dendrobium officinale* could be used to distinguish the plant from different sources. These findings facilitate a better understanding of the phytochemical variation of *Dendrobium officinale*, contributing to better quality control.

### 2.2. Influential Factors

The varied growth environment and origins with different natural resources lead to significant differences in the yield, quality, and even medicinal values of *Dendrobium officinale*. Samples from Zhejiang, Fujian, Yunnan, and Jiangxi Provinces resulted in different compositions of the active compounds, of which only the sample from Yunnan Province had three unique medicinal components, and only the sample from Jiangxi Province had no toxic component [26]. Hence, *Dendrobium officinale* from different regions need more investigations for better collection, protection, and utilization.

The quality and biological activities of *Dendrobium officinale* could be influenced by the processing methods and storage conditions [27–29]. The reduction in the grinding particle size could result in better physical properties and higher solubility of protein and polysaccharides, which led to better bioavailability and stronger antioxidant activity than crude ground products [27]. In addition, heat might cause the decrease and destruction in polysaccharides of glucomannan-rich and glucan-rich samples, and thus, lyophilization and torrefaction rather than the traditional dry method could better retain the polysaccharides and preserve their best nutritional value [6]. Moreover, the extraction method could affect the rheological and physicochemical properties of polysaccharides from *Dendrobium officinale*, such as mannanase activity, carbohydrate content, hydrophobicity, and viscosity [29]. And it further influences the composition of fractions, including the molecular weights and molar ratios of *D*-mannose and *D*-glucose, leading to different degrees of biological activities [30]. The freeze-thawing cold-pressing could extract polysaccharides with high yield, well-preserved form, and strong antioxidant activity, compared to conventional extraction methods like hot water extraction, cold-pressing, and ultrasonic-, microwave- and enzyme-assisted hot water extraction [31]. On the other hand, during the storage, the low temperatures could induce an increase in polysaccharide content and higher antioxidant activity than the ambient temperature, while the starch content was decreased. It indicated that storing the postharvest *Dendrobium officinale* at low temperatures could lead to higher levels of polysaccharides and longer shelf-life [32].

In short, the quality and bioactivities of *Dendrobium officinale* are susceptible to many factors like processing methods, extraction methods, and storage conditions, and thus, it is necessary to choose proper procedures to control and increase the quality of raw material and products of *Dendrobium officinale*.

## 3. Bioactive Properties

### 3.1. Antioxidant Activity

The excessive production of reactive oxygen species (ROS) could disrupt the balance of the antioxidant defense system and cause oxidative stress which works as a component of many diseases, including cardiovascular diseases, Alzheimer's diseases, and cancer. *Dendrobium officinale* and its bioactive components showed potent antioxidant activity and attenuated oxidative stress-induced injuries. The polysaccharides (250 and 500 *μ*g/mL) from *Dendrobium officinale* could protect the human gastric mucosal epithelial cells against H_2_O_2_-induced apoptosis by decreasing the level of ROS and improving the nuclei morphology. Additionally, the animal model further confirmed that polysaccharides attenuated the gastric mucosal injury and reduced the oxidative stress-induced apoptosis by downregulating the ratio of Bcl-2-associated X (Bax)/B-cell lymphoma-2 (Bcl-2) protein expression in gastric mucosa [33]. Moreover, *Dendrobium officinale* could activate nuclear factor erythroid 2-related factor 2 (Nrf2) signaling and the antioxidant enzymes to mitigate the damage induced by ROS. The treatment of polysaccharides at a high dose of 9.6 g/kg could protect against the precancerous lesions of gastric cancer (PLGC) in rats by activating the Nrf2 pathway and its downstream antioxidant enzymes like heme oxygenase 1 (HO-1) and NAD(P)H: quinone oxidoreductase-1 (NQO-1). The treatment also reduced the levels of 8-hydroxy-deoxyguanosine (8-OHdG) which was one of the predominant biomarkers of free radical-induced oxidative stress [34, 35]. In addition, polysaccharides could reduce oxidative stress-induced injuries by elevating the activity of the antioxidant enzyme superoxide dismutase (SOD) and decreasing the level of malonaldehyde (MDA), a product of polyunsaturated fatty acid peroxidation, in rats with type 2 diabetes treated at the dose of 20, 40, 80 and 160 mg/kg b.w. [36]. Like other natural products, *Dendrobium officinale* could also work as a dietary antioxidant supplement. However, its induction of antioxidant defenses may fail to reach effective concentration and the significant effects on human study. More importantly, some progression of diseases might be attributed to oxidative stress as the secondary contributor instead of the primary cause, and thus, the antioxidant properties of *Dendrobium officinale* may not pose a significant influence on the diseases [37].

### 3.2. Anti-inflammatory Activity

Chronic inflammation is a vital risk factor for various diseases such as diabetes, cancer, and cardiovascular diseases, and thus, the effective inhibition of inflammation facilitates the control and prevention of many chronic diseases. The in vivo and in vitro studies revealed that *Dendrobium officinale* and its bioactive compounds could inhibit inflammation by modulating inflammatory cytokines and related mediators. Sjogren's syndrome is a chronic autoimmune disorder of the affected glands with lymphocytic infiltration and dysfunction of aquaporin 5 (AQP5). A clinical study was conducted with 16 female patients with the deficient secretion of saliva, and they randomly received the extracts at the dose of 0.5 g/5 mL three times daily for one week. The results revealed that the treatment improved the function of glands by regulating the expression of AQP5 in labial glands and increasing saliva secretion [38]. The mouse model with Sjogren's syndrome further demonstrated the underlying mechanism that *Dendrobium officinale* polysaccharides (20 mg/mL) could reduce the expression of proinflammatory cytokines like tumor necrosis factor-alpha (TNF-*α*), interleukin-1 beta (IL-1*β*), and IL-6, which attenuated the immune-mediated inflammation and maintained the balance of inflammatory cytokines [39]. Additionally, the pretreatment of polysaccharides at the dose of 1 *μ*g/mL could inhibit the TNF-*α*-induced apoptosis of human salivary gland cell line A-253 cells, indicating its potential of protecting the salivary glands and ameliorating Sjogren's syndrome [40]. Polysaccharides (1.5 g/kg) reduced brain inflammation and seizures, which were mainly involved in inhibiting the expressions of IL-1*β* and TNF-*α* as well as mitogen-activated protein kinase (MAPK) signaling pathways in pentetrazol-induced epileptic rats [41]. Moreover, the polysaccharides isolated from the leaves could also mitigate the inflammation in LPS-stimulated THP-1 cells, and it could protect the cells against cytotoxicity and reduce the formation of ROS, which may be associated with the inhibition of TLR-4, myeloid differentiation factor (MyD88), and tumor necrosis factor receptor-associated factor-6 (TRAF-6) [42].

### 3.3. Immune-regulatory Activity

*Dendrobium officinale* and its bioactive compounds have been reported to have the capability of regulating the immune system via cytokines and immune cells. For instance, 25 *μ*g/mL of the purified polysaccharides stimulated the immune activities by activating the extracellular signal-regulated kinases 1/2 (ERK1/2) and NF-*κ*B signaling pathways in human leukemia monocytic cell line THP-1 cells [43]. In addition, the treatment of 2,3-*O*-acetylated-1,4-beta-*D*-glucomannan (100 *μ*g/mL) could target chemotactic cytokines like chemokine (C-C motif) ligands 4 (CCL4) and interferon gamma-induced protein 10 (IP-10) to stimulate the immune response in THP-1 cells, and these effects were mainly associated with the activation of NF-*κ*B which was regulated through the Toll-like receptor 4 (TLR4) signaling pathway [44].

The immune cells are also affected by the treatment of *Dendrobium officinale*. The purified polysaccharide with the main structure of *O*-acetyl-glucomannan at the dose of 40, 80, and 160 mg/kg b.w. promoted the proliferation of splenocytes; regulated the spleen lymphocyte subsets; increased the levels of serum immunoglobulin M (IgM), IgG, and haemolysin; and improved the phagocytotic function in cyclophosphamide-induced immunosuppressed mice [45]. Moreover, some subfractions of polysaccharides exhibited immunomodulating activity and enhanced the immune response by increasing the proliferation of splenocytes and macrophages, the secretion of cytokines like TNF-*α*, and the production of NO as well as phagocytosis [46–48]. In addition, the treatment of *Dendrobium officinale* polysaccharides at the dose of 1 *μ*g/mL in Sjogren's syndrome model could reduce the abnormal infiltration and apoptosis of lymphocytes, attenuate the dysfunction of AQP5, and induce the translocation of AQP5 by activating M3 muscarinic receptors, which indicated its ability to improve the immunity [39, 49].

Moreover, the immune-regulatory activity of *Dendrobium officinale* is closely associated with gut microbiota. The feeding of 0.25% polysaccharides increased the abundance of the gut microbiota *Parabacteroides*, in which *Parabacteroides*_sp_HGS0025 was positively associated with the butyrate, IgM, IL-10, and interferon-alpha (IFN-*α*) in the intestine and blood of mice. It indicated that polysaccharides could improve immunity by regulating the intestinal microbiota and its metabolites like butyrate [50].

## 4. Health Benefits

### 4.1. Anticancer Effects

*Dendrobium officinale* has therapeutic potential in cancer prevention and treatment. Its potential mechanism of action is mainly involved in reducing cancer cell growth and proliferation, triggering apoptosis, and increasing autophagy. And therefore, the adjuvant use of *Dendrobium officinale* might be utilized as a simple, safe, but feasible therapy for cancer treatment.

Cancer cells have little apoptosis, and they could shift to malignant cells for lasting existence, induce tumor metastasis, and increase resistance to anticancer drugs [51]. The polysaccharides from *Dendrobium officinale* had the capability of triggering apoptosis to limit cancer progression. The apoptosis of cancer cells is mainly mediated by the antiapoptotic and proapoptotic cytokines and pathways. The well-known antiapoptotic factors include proteins like Bcl-2, Bcl-extra large (Bcl-xL), and Mcl-1, while the proapoptotic factors involve proteins like Bax, Bcl-2 homologous antagonist killer (Bak), Bcl-2 interacting killer (Bik), p53, and caspase-3 [52]. A study found that the polysaccharide extracted by hot water was effective in dose-dependently inhibiting the growth of liver hepatocellular carcinoma cell line HepG2 cells by increasing the ROS level, decreasing mitochondrial membrane potential, and inducing apoptosis with the downregulation of antiapoptotic protein Bcl-2 and upregulation of proapoptotic protein Bax expressions [53]. In addition, the polysaccharides effectively inhibited the proliferation of osteosarcoma U2OS and Saos-2 cells. It had a synergistic effect with cisplatin, which increased the cisplatin-induced apoptosis by upregulating the expression of proapoptotic factors p53, Bax, and Bak, downregulating the expression of antiapoptotic factors Bcl-2 and Mcl-1, and increasing the ratios of cleaved caspase-9 to caspase-9, cleaved caspase-3 to caspase-3, and cleaved poly (ADP-ribose) polymerase (PARP) to PARP [54]. Moreover, after being degraded into smaller molecules, the fractions of polysaccharides exerted inhibitory effects on the proliferation of human cervical carcinoma HeLa cells and induced apoptosis by upregulating the expression of ERK, Jun N-terminal kinase (JNK), and p38 [12].

The regulation of the Wnt signaling pathway is also closely associated with tumorigenesis. PLGC is a major phase in the progression of gastric cancer, which might be a potential target for the treatment of gastric cancer. The polysaccharides prepared from *Dendrobium officinale* were found to ameliorate the MNNG-PLGC in rats via the Wnt/*β*-catenin pathway, downregulating the expressions of Wnt2*β* and glycogen synthase kinase 3 beta (Gsk3*β*), proliferating cell nuclear antigen (PCNA), and cyclinD1. In addition, the results of serum endogenous metabolites revealed that the change in betaine was the most significant, indicating that betaine may be a key contributor to the anticancer effects of *Dendrobium officinale* polysaccharides [55].

Furthermore, mitochondria are considered important in apoptosis, and a high level of ROS generated from mitochondria could induce apoptosis in cancer cells, suggesting that targeting the mitochondrial function or ROS stimulation might be feasible in cancer cell treatment [56]. An in vitro study with colon cancer cell line CT26 cells showed that the polysaccharides isolated from *Dendrobium officinale* reduced the proliferation of cells and induced cytotoxic autophagy as well as mitochondrial dysfunction via the ROS-AMP-activated protein kinase- (AMPK-) autophagy pathway [57].

On the other hand, *Dendrobium officinale* could prevent the growth of tumors by improving the host function and responses. For instance, the polysaccharide of *Dendrobium officinale* reduced colon tumorigenesis by preserving the intestinal barrier function and improving immune response to the tumor microenvironments in mice with colorectal cancer. The intestinal barrier function was restored by increasing the expression of zonula occludens-1 (ZO-1) and occludin, and the immune response was increased to exert anticancer effects via the tumor infiltrated CD8(+) cytotoxic T lymphocytes (CTLs) and the expression of programmed death-1 (PD-1) on CTLs [58].

The potential anticancer activity of *Dendrobium officinale* could potentiate the efficacy of anticancer agents or chemotherapy. Its polysaccharides inhibited the growth of human colorectal cancer HT-29 cells and reduced the metastasis of tumors in the zebrafish model, and the treatment increased the anticancer efficacy of 5-fluorouracil, which induced apoptosis via the mitochondrial-dependent intrinsic apoptotic pathway. These results indicated that *Dendrobium officinale* is a potential candidate for colorectal cancer therapy alone or in the combination with chemotherapy medication [59].

Notably, the molecular weight and structure of compounds in *Dendrobium officinale* might influence its anticancer activities. A study compared the anticancer properties of carbohydrates in *Dendrobium officinale* with different molecular weights, including monosaccharides, oligosaccharides, and polysaccharides. It was reported that polysaccharides had better anticancer properties than monosaccharides and oligosaccharides, suggesting that the efficacy of carbohydrate drugs largely depends on the molecular weight of the cancer treatment [54]. Moreover, the modification and use of vehicles could promote bioavailability and increase the bioactive function of *Dendrobium officinale*. The gold nanoparticle synthesized from the extracts of *Dendrobium officinale* showed better anticancer effects without increasing toxicity to the host [60]. In addition to the polysaccharides, there are also new derivates from *Dendrobium officinale* with significant anticancer activities. A study found that several new phenanthrene and 9,10-dihydrophenanthrene derivative compounds showed cytotoxicity against cancer cell lines, HI-60 and THP-1 cells, and one of the compounds had a most significant effect with IC50 values of 11.96 and 8.92 *μ*M, respectively [61].

### 4.2. Antidiabetic Effects

Diabetes mellitus is a metabolic disorder and global health concern with complicated factors. The rapid development of modern society leads to unhealthy eating behavior, less physical activities, and overloaded stress management, which increases the risk of diabetes in adolescents and young adults [62]. In addition, the occurrence of diabetes increases the risk of complications that are still costly to be controlled by current drugs, such as diabetic retinopathy and nephropathy [63]. Numerous studies reveal that many herbal medicines and their bioactive compounds show significant hypoglycemic effects mainly by regulating glucose metabolism, improving insulin sensitivity and insulin resistance, and restoring the damaged pancreas [64]. *Dendrobium officinale* as a medicinal herbal plant has a long history of being used to attenuate the symptoms of diabetes which is also called “Xiaoke” disease in China. The hypoglycemic efficacy of *Dendrobium officinale* makes it a common ingredient in Xiaoke decoction for type 2 diabetes treatment [65].

Some enzymes are involved in glycemic control, such as *α*-glucosidase and *α*-amylase, and they have been developed as therapeutic targets for type 2 diabetes prevention and treatment [66]. Several main antidiabetic compounds were identified based on the inhibition of *α*-glucosidase and *α*-amylase activities in *Dendrobium officinale*. The crude extract of its stems was reported to have IC50 values of 78.1 *μ*g/mL on *α*-glucosidase activity and 116.7 *μ*g/mL *α*-amylase activity. Moreover, there were six compounds associated with *α*-glucosidase inhibition, such as N-p-coumaroyltyramine and 3,4,4′-trihydroxy-5-methoxybibenzyl. And 3,4-dihydroxy-4′,5-dimethoxybibenzyl was the only identified compound with *α*-amylase inhibitory activities [67]. Type 2 diabetes is tightly related to abnormal metabolisms, such as hepatic glucose metabolism, insulin resistance, and low-grade inflammation. *Dendrobium officinale* polysaccharides could decrease the levels of fasting blood glucose, insulin, glycated serum protein, and serum lipid profile and alleviate pancreatic injury as well as the dysregulated metabolism of bile acids and amino acids in type 2 diabetic rats [36]. In addition, it could regulate the hepatic glucose metabolism via the glucagon-mediated signaling pathways as well as the liver-glycogen structure in HFD/STZ-induced diabetic mice [68]. Furthermore, the polysaccharides reduced the fasting blood sugar levels in mice by increasing insulin in serum and stimulating the glucagon-like peptide-1 (GLP-1) secretion which is an important hormone regulator in the progression of diabetes. And the in vitro study showed that the stimulated GLP-1 secretion may be related to the Ca^2+^/calmodulin-dependent protein kinase (CaMK) and p38-MAPK pathways in the murine enteroendocrine cell line STC-1 cells [69]. *Dendrobium officinale* extracts could prevent STZ-induced type 1 diabetes in mice, which increased the level of liver glycogen and taurine and upregulated energy and amino acid metabolism [70].

Apart from type 1 and type 2 diabetes, diabetic complications are also recognized as a severe health concern. *Dendrobium officinale* polysaccharides were demonstrated to ameliorate diabetic cataracts in rats, and it could reduce the severity of the opacity of the lens by downregulating the microRNA-125b and MAPK signaling pathways, in which the level of microRNA-125b was positively correlated with the levels of ERK1, ERK2, Raf, and Ras [71].

### 4.3. Gastrointestinal Modulation

In the past two decades, numerous findings have revealed that the gut microbiota and its derived microbial products are key influential factors in the host metabolism, and dysbiosis is tightly linked to a high risk of many metabolic diseases [72]. The polysaccharides of *Dendrobium officinale* could regulate the composition and abundance of gut microbiota and its metabolites in mice, which increased the beneficial bacterium like *Ruminococcus*, *Eubacterium*, *Clostridium*, *Bifidobacterium*, *Parabacteroides*, and *Akkermansia muciniphila* and decreased the harmful bacteria like *Proteobacteria* and further modulated the production of butyrate [50]. In addition, *Dendrobium officinale* increased the diversity of intestinal mucosal flora in mice fed with HFD, which enhanced the abundance of *Ochrobactrum* and reduced the abundance of *Bifidobacterium* and *Ruminococcus*, and it further influenced the metabolism of carbohydrate, energy, and amino acid as well as gut microbiota to reduce HFD-induced negative effects [73].

As the most abundant and common microbial metabolites, short-chain fatty acids (SCFAs) play an important role in the gut and metabolic health. Studies found that SCFAs mediated the G-protein coupled receptors (GPCRs), such as GPCR41 and GPCR43, and the regulation of the SCFA-GPCR pathway by *Dendrobium officinale* could alleviate metabolic disorders [74, 75]. Moreover, the enzymatic fragments of polysaccharides could protect against dextran sulfate sodium- (DSS-) induced colitis by ameliorating the gut microbiota dysbiosis. The treatment inhibited the proinflammatory cytokines, restored SCFA levels, increased GPCR levels, and regulated the gut microbiota, which increased the abundance of *Bacteroides*, *Lactobacillus*, and *Ruminococcaceae* and reduced the abundance of *Proteobacteria* [74]. On the other hand, the polysaccharides were found little absorbed and would be degraded into SCFAs in the large intestine after the oral administration, and thus, its modulatory effects on gut microbiota were considered the main contributor to its bioactivities [76].

**Figure 2:**
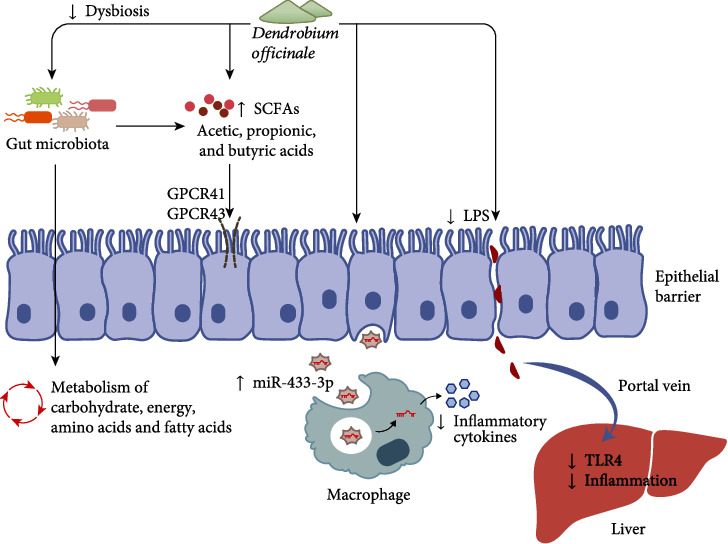
The gastrointestinal modulatory activities of *Dendrobium officinale* via several pathways.

*Dendrobium officinale* could not only alleviate metabolic disorders via the modulation of intestinal microbiota and microbial products but also improve gut health to maintain host homeostasis (Figure 2). An in vitro fermentation study showed that polysaccharides from *Dendrobium officinale* increased the levels of SCFAs which mainly contained the acetic, propionic, and butyric acids, and it changed the gut microbiota community and accelerated the metabolic pathways of amino acid and fatty acids. The results suggested that the polysaccharides had probiotic effects improving gastrointestinal health [77]. On the other hand, the polysaccharides could ameliorate inflammatory bowel disease by increasing miR-433-3p in the intestinal small extracellular vesicle. The increased delivery of miR-433-3p reduced the inflammation from excessive macrophage activity in the intestine by inhibiting the MAPK signaling pathway, which was beneficial for maintaining the intestinal microenvironment [78]. Besides, the *O*-acetyl-glucomannan extracted from *Dendrobium officinale* was found to improve the colonic microenvironment and benefit colon health in mice, which increased the content of SCFAs, colonic length, and fecal moisture and reduced the colonic pH and defecation time [79]. Furthermore, the ethanol-induced gastric mucosal injury could be protected by the polysaccharides from *Dendrobium officinale* leaves consisting of mannose, galacturonic acid, glucose, galactose, and arabinose, and it could improve antioxidant capacity and reduce the apoptosis in human gastric epithelial cell line GES-1 cells via the AMPK/mTOR signaling pathway [80].

The gut-liver axis has attracted great attention in the field of liver diseases since the gut-derived products could be transported directly to the liver via the portal vein, and the liver could give feedback via the bile and antibody secretion to the intestine [81]. After the mice were withdrawn from the high sugar and high-fat diet, *Dendrobium officinale* accelerated the liver recovery and inhibited the lipid deposition as well as inflammatory lesions in the liver, which was involved in modulating the gut microbiota and suppressing the activation of LPS-TLR4-associated inflammatory mediators in mice with NAFLD [79]. However, little is known about the underlying mechanism of action, and it is necessary to shift from the descriptive interaction analysis between the treatment of *Dendrobium officinale* and gut microbiota composition to cause-and-effect studies. And more microbiota-targeted interventions could be conducted to improve metabolic health in humans.

### 4.4. Cardiovascular Protection

Cardiovascular diseases remain a major threat to public health and human life, and it is caused by various pathological factors such as oxidative stress and inflammation [82]. It has been reported that *Dendrobium officinale* exerted cardiovascular-protective effects mainly by defending against oxidative stress, reducing the apoptosis of cardiomyocytes, and suppressing inflammation. The polysaccharides of *Dendrobium officinale* protected cardiomyocytes against oxidative stress-induced apoptosis by reducing ROS production, restoring mitochondrial membrane potential, regulating apoptosis-related protein, and increasing the activity of antioxidant enzymes, and these effects were possibly associated with the regulation of phosphoinositide 3-kinases (PI3K)/Akt and MAPK pathways [82, 83]. Moreover, *Dendrobium officinale* extracts had protective potential against diabetic cardiomyopathy in STZ-induced diabetic mice, which inhibited oxidative stress, decreased cardiac lipid accumulation as well as deposition of collagen, downregulated the expression of several proinflammatory cytokines, and reduced cardia fibrosis [84]. Furthermore, *Dendrobium officinale* could ameliorate the aberrant cardio condition through the regulation of metabolism. In the rat model of unhealthy diet-induced metabolic hypertension, *Dendrobium officinale* could alleviate hypertension by reducing lipid abnormalities and improving the function of gastrointestine as well as vascular endothelial relaxation, which may be mediated by activating the SCFA-GPCR 43/41 pathway [75, 85]. Besides, the water-soluble extracts of *Dendrobium officinale* alleviated cardiac injury and fibrosis in HFD/STZ-induced diabetic mice with a 12-week daily administration, which was potentially implicated in increasing lipid transport, reducing insulin resistance, and inhibiting the EMT signaling pathway [86].

### 4.5. Liver Protection

*Dendrobium officinale* could also confer protection against liver injuries and improve liver functions against different forms of liver injuries, such as drug-, chemical-, and acute alcohol-induced injuries and nonalcoholic fatty liver diseases (NAFLD). The polysaccharides from *Dendrobium officinale* could attenuate the acetaminophen-induced hepatotoxicity in mice by reducing the oxidative stress and activating the Nrf2-Keap1 signaling pathway, in which the levels of alanine aminotransferase (ALT), aspartate aminotransferase (AST), ROS, MDA, and myeloperoxidase (MPO) were decreased; the levels of GSH, CAT, and T-AOC were increased, and the Nrf2 nuclear translocation was activated [87]. Additionally, alcoholic liver diseases are characterized by disrupted ethanol metabolism and stimulated oxidative stress. The NIR fluorescence imaging showed that the polysaccharides from *Dendrobium officinale* could protect against acute alcoholic liver injury in vivo by increasing the antioxidant levels, in which the level of GSH was balanced in the liver [88]. In addition, the alcohol-induced liver injury could be mitigated by the extracts of the *Dendrobium officinale* flower, which was associated with its antioxidant and anti-inflammatory activities. The flower extracts treatment reduced the serum levels of ALT, AST, TC, and TG and increased the activities of the antioxidant enzymes. It was associated with the downregulation of hepatic cytochrome P450 2E1 (CYP2E1) and upregulation of Nrf2, HO-1, and NQO1. Moreover, it inhibited inflammation by downregulating TLR-4 and NF-*κ*B p65 [89].

NAFLD is often caused by excessive lipid accumulation or steatosis due to an unhealthy diet pattern with little or no alcohol consumption. After the high-sucrose and high-fat diet was stopped, the 3-week administration of *Dendrobium officinale* could reduce the hepatic lipid accumulation, regulate the metabolism of fatty acid, and improve the histopathology of the liver in NAFLD mice. It may increase the *β*-oxidation and reduce the synthesis, desaturation, and uptake of fatty acids and alleviate the abnormality of major phospholipids in the liver of mice [90]. Furthermore, the polysaccharides could also reduce the disturbed hepatic lipid metabolism involved with the fatty acid, glycerolipid, and glycerophospholipid, and it restored the metabolism of ceramide and bile acids in type 2 diabetic rats [85].

### 4.6. Lung Protection

Due to climate changes and personal unhealthy lifestyles, the risk of chronic respiratory disease and acute lung injury goes high in these decades. Chronic obstructive pulmonary disease increases airway inflammation and leads to respiratory dysfunction. Cigarette smoke is a vital risk factor for the incidence of chronic obstructive pulmonary disease, and it could induce mucus hypersecretion and viscosity. Both in vitro and in vivo studies found that *Dendrobium officinale* polysaccharides attenuated the cigarette smoke-induced mucus hypersecretion and viscosity by inhibiting mucus secretory granules and downregulating the expression of mucin-5AC (MUC5AC) [91]. Moreover, a randomized, double-blind, and placebo-controlled clinical trial was conducted on 40 patients with smoking habits and mild airflow obstruction, and patients randomly received 1.2 g *Dendrobium officinale* polysaccharides thrice daily. The treatment of polysaccharides could significantly ameliorate lung functions and reduce the serum levels of proinflammatory mediators (IL-6, IL-8, CRP, and TNF-*α*), and the expression of MUC5AC was decreased, and AQP5 was increased [92]. Additionally, it could decline cigarette smoke-induced oxidative stress in the lung and decrease the number of lymphocytes as well as monocytes in serum, which reduced the infiltration of inflammatory cells in lung tissue and inflammation indicators in serum. These effects might be mediated by inhibiting MAPK and NF-*κ*B signaling pathways [93].

The polysaccharides of *Dendrobium officinale* attenuated the bleomycin-induced pulmonary inflammation and fibrosis in rats by inhibiting the transforming growth factor-beta 1- (TGF-*β*1-) Smad2/3 signaling pathway, and it effectively suppressed the transformation of alveolar epithelial type II cells into myofibroblasts and reduced the expression of Smad2/3 and fibronectin in rats [94]. Besides, colitis-induced secondary lung injury could be attenuated by the polysaccharides, which reduced inflammation and oxidative stress. The treatment inhibited the protein expression of TLR4 and increased the protein expressions of Nrf2, HO-1, and NQO-1 both in mice and in LPS-stimulated BEAS2B cells, indicating that TLR4 and Nrf2 signaling pathways played an important role in it [95].

### 4.7. Neuroprotection

*Dendrobium officinale* plays a crucial role in protecting the nervous system potentially by reducing neurological damage and improving memory as well as cognitive function. The extracts of *Dendrobium officinale* reduced the impaired neurobehaviors and enhanced the antioxidant capacity in neonatal rats with hypoxic-ischemic brain damage (HIBD), and it protected against HIBD by inhibiting neuronal apoptosis and increasing the expression of neurotrophic factors [96]. Additionally, its polysaccharides could attenuate learning and memory disabilities in mice, and these effects may be mediated by regulating the Nrf2/HO-1 pathway and inhibiting the activation of astrocytes and microglia in cognitive decline [97]. Moreover, the flower of *Dendrobium officinale* was found to attenuate the depression-like behavior in mice with the increase in sucrose consumption and decrease in immobile time, which may be mediated by the increased expression of nerve growth factor (NGF) and brain-derived neurotrophic factor (BDNF) in hippocampus. And the regulation of neurotrophic factor expression was also verified in astrocytes through a cAMP-dependent mechanism, plasminogen, and MMP-9 [98]. However, the capacity to cross the blood-brain barrier determines the direct action of natural products in the central nervous system. The extracts of *Dendrobium officinale* may contain complex biomacromolecules that fail to cross the blood-brain barrier, and thus, they may influence brain functions via some indirect pathways like the gut-microbiota-brain axis after oral intake [99].

### 4.8. Other Health Benefits

There are also other bioactivities and health benefits of *Dendrobium officinale*. Its polysaccharide has confirmed its antiosteoporosis activity through increasing osteogenic differentiation of bone marrow mesenchymal stem cells (BMSCs) and reducing adipogenic differentiation. The in vitro study revealed that the polysaccharides restored the H_2_O_2_-induced abnormal cell differentiation, while the in vivo study showed that it increased the bone mass and reduced the marrow adipose tissue as well as the oxidative stress in the aged mice, in which the activation of the Nrf2 antioxidant signaling pathway was considered the main contributor to these effects against age-related osteoporosis [100]. It also ameliorated the ovariectomy- and receptor activator expression of the NF-*κ*B ligand- (RANKL-) induced osteoporosis by improving the bone microarchitecture, preventing bone loss, inhibiting osteoclastogenesis, and reducing the expression of osteoclast-specific markers [101].

Moreover, *Dendrobium officinale* and its bioactive compounds exert potent antifatigue effects. The 4-week treatment of polysaccharides with glucomannan in size of 730 kDa could ameliorate the fatigue in mice and reduce the indicators of fatigue, such as the increased levels of lactic dehydrogenase (LDH), blood urea nitrogen (BUN), MDA, creatine phosphokinase (CK), and lactic acid (LD) and the decreased levels of serum SOD/glutathione peroxidase (GSH-Px) and gastrocnemius glycogen [102]. Also, the extracts attenuated fatigue and improved fatigue resistance of mice by maintaining the glycogen storage, reducing oxidative stress, and promoting the expression of peroxisome proliferator-activated receptor-gamma coactivator 1-alpha (PGC-1*α*) [103].

In addition, *Dendrobium officinale* showed potential antiobesity activity. The polysaccharides could reduce palmitic acid-induced insulin resistance in vitro by activating the expression of peroxisome proliferator-activated receptor-gamma (PPAR-*γ*). It also declined the abnormal lipid metabolism and reduced the inflammation of visceral adipose tissue in both diets and genetically induced obese mouse models [104]. Furthermore, both the ultrafine powder and polysaccharides with glucose and mannose (14 : 1) exerted laxative activity and alleviated constipation by improving the colonic motility function, increasing gastrointestinal transit ratio, and regulating the gut hormones like motilin, gastrin, acetyl cholinesterase, substance P, and somatostatin [1, 105].

**Figure 3:**
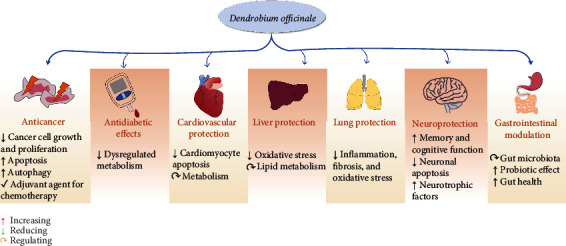
A summary of the health benefits of *Dendrobium officinale*.

As mentioned above, a growing body of evidence indicates that as a traditional medicine and food homologous plant, *Dendrobium officinale* has diverse biological properties and health benefits (Figure 3). The bioactivities and related mechanism of actions of *Dendrobium officinale* extracts are shown in Table 1, while that of its polysaccharides is summarized in Table 2.

## 5. Safety

With the widespread usage and consumption of *Dendrobium officinale*, it is essential to assure its safety and quality from aspects of cultivation, preparations, and storage. Although there is a risk for herbal plants to be contaminated by heavy metals and pesticide residue, it is often safe to consume within a certain dose range [106]. A total of 43 different pesticides were found in *Dendrobium officinale* samples from three different growing regions, of which dimethomorph was the highest one. But the risk assessment demonstrated that there was no potential exposure risk of pesticides in *Dendrobium officinale* to human health in both the long and short terms [3]. In addition, the analysis of liquid chromatography-tandem mass spectrometry with 12 pesticides showed that the half-lives of pesticides were 0.9-14.4 days, and trifloxystrobin and fluopyram required the longest interval to harvest (42 days). The chronic and acute risk assessment data illustrated that the residues of these 12 pesticides in *Dendrobium officinale* posed no harmful effect on human health. The chronic and acute risk quotients of common pesticides were quite low, indicating that *Dendrobium officinale* showed little toxicity as dietary consumption in the general population [107]. There was little report about the significant toxicity induced by the consumption of *Dendrobium officinale*. On the other hand, the daily intake should not exceed 12 g according to *Chinese Pharmacopoeia* (2020 Edition), and it is not recommended for pregnant and lactating women and infants. More clinical studies are in demand for the risk assessment of humans under exposure to *Dendrobium officinale*.

In short, *Dendrobium officinale* is a relatively safe herbal product with high edibility and various bioactivities. Apart from controlling the safety and quality during plantation, processing, and storage, it is still essential to manage the consumption within an effective but safe dosage and proper duration for patients as a therapeutical agent or dietary supplement.

## 6. Challenges and Outlooks

Although *Dendrobium officinale* might be a potential candidate for dietary supplements in disease treatment, some challenges are needed to be considered in future work. As a traditional Chinese herbal medicine, *Dendrobium officinale* is often used in combination with other herbal medicines as complicated formulations. Thus, the relationship between major active compounds and diseases remains vague, and the synergistic therapeutic effects of formulations might complicate the investigation of the mechanism of individual ingredients [108]. In the last decade, increasing studies have utilized computational methods to explore this complex interaction, such as network pharmacology and bioinformatics, which could establish the model of “compound-protein/gene-disease” via databases to identify the role of certain compounds in disease treatment and predict the therapeutical targets [109]. Many studies on traditional Chinese medicine have used high-throughput transcriptomic screening for investigating the molecular effects of herbs or ingredients, which might help explore novel molecular mechanisms and support the modernization of herbal medicines and herb-derived drug discovery [110].

Most studies have concentrated on the crude polysaccharides *of Dendrobium officinale*, but their bioactivities are closely associated with the structure features, molecular weight, and ratio of components like galactose, glucose, and mannose [111]. The alkali-soluble polysaccharide from *Dendrobium officinale* showed better effects on the proliferation of lactic acid bacteria and Bifidobacteria during the fermentation than the water-soluble polysaccharide, which is mainly attributed to its higher level of total sugar, uronic acid, glucose, and mannose as well as the lower level of sugar [112]. Hence, more attention should be paid to investigations of structure-activity relationships of *Dendrobium officinale* polysaccharides [113]. On the other hand, more efforts are now being made on the identification and characterization of the structural features and compositions of *Dendrobium officinale* polysaccharide fractions, but few of them have been standardized and developed as individual ingredients or drugs for extensive pharmacological research, which might hinder the definition of mechanism and clinical application. Additionally, the quality of *Dendrobium officinale* is susceptible to multiple factors like the cultivation origin, processing, and storage procedures. In particular, the processing methods are used to extract and purify *Dendrobium officinale*, and it might result in the modification of the chemical structure or degradation of active compounds, negatively affecting their bioavailability.

Moreover, like most herbal medicines, the pharmacokinetic, absorption, distribution, metabolism, and excretion studies of *Dendrobium officinale* are rarely documented, and the present pharmacokinetic studies mainly concentrated on several herbal medicines, like curcumin, ginseng, and ginger [114, 115]. However, these studies are essential for modern drug development and clinical application. The effective dose levels, tissue distribution, and metabolites of *Dendrobium officinale* are important elements for its bioactivities and action targets, which should be further analyzed by pharmacokinetic, absorption, distribution, metabolism, and excretion studies [116].

Although *Dendrobium officinale* has a long history of being used as formulations in folk, clinical study about its individual effects on human health is still scarce and limited. More detailed and large-scale clinical trials are warranted to assess its bioactivities and therapeutical effects on different diseases.

## 7. Conclusion

*Dendrobium officinale* has been widely used as a functional food and herbal medicine for preventing and managing many disorders. The phytochemical studies showed that *Dendrobium officinale* contains abundant bioactive compounds, such as bibenzyls, polysaccharides, flavonoids, and alkaloids. The experimental investigations revealed that *Dendrobium officinale* exerted antioxidant, anti-inflammatory, and immune-regulatory properties. It had a diversity of pharmaceutical effects like anticancer, antidiabetes, gastrointestinal modulatory, cardiovascular protective, hepatoprotective, lung protective, and neuroprotective activities. Hence, *Dendrobium officinale* could be considered the potential agent of adjuvant supplements for disease treatment. However, most studies focused on crude polysaccharides as the major medicinal compound, and few new components were purified for investigations. Although *Dendrobium officinale* has been used for a long time in folk, detailed and large-scale clinical studies are still warranted to demonstrate the pharmacological effects and mechanisms in humans. In addition, more investigations combining different modern technologies are needed for better control of the quality and safety of *Dendrobium officinale*.

## Figures and Tables

**Table 1:** The health benefits and related molecular mechanisms of *Dendrobium officinale* extracts.

Type of study	Object	Dosage	Effects	Potential mechanisms	References
*Antidiabetes*					
In vivo	Male STZ-induced diabetic C57BL/6 mice	300 and 700 mg/kg b.w.	Decreased the levels of blood glucose Increased the levels of glycogen in liver Upregulated the energy and amino acid metabolism	↑ Citrate, pyruvate, alanine, isoleucine, histidine, and glutamine in serum ↑ Alanine and taurine in liver	[70]
In vivo	Male STZ-induced type 1 diabetic Sprague-Dawley rats	1 g/kg b.w.	Decreased the levels of serum TC, TG, BUN, and CREA Attenuated the hypoalgesia and histopathological changes of vital organs induced by hyperglycemia Prevented early complications in type 1 diabetes	↑ GSH-Px	[117]
*Gastrointestinal modulation*					
In vivo	Male and female Kunming mice	2.37 g/kg b.w.	Increased the diversity of intestinal mucosal flora Changed the carbohydrate, energy, and amino acid metabolism of intestinal mucosal flora Reduced the negative effects induced by HFD	↑ *Ochrobactrum* ↓ *Bifidobacterium* and *Ruminococcus*	[73]
In vivo	Male ICR mice	0.2 and 0.6 g/kg b.w.	Regulated the gut microbiota Prevented lipid deposition and inflammatory lesions in the liver Inhibiting LPS-TLR4-associated inflammatory mediator activation Accelerated liver recovery	NA	[79]
*Cardiovascular protection*					
In vivo	Male Kunming mice	75, 150, and 300 mg/kg b.w.	Protected against myocardial ischemia Reduced the infarct size and the number of apoptotic cardiomyocytes	↑ SOD ↑ Meis1 ↓ CK-MB and LDH ↓ MDA	[118]
In vivo	Male STZ-induced diabetic Kunming mice	75, 150, and 300 mg/kg b.w.	Decreased the ratio of heart to body weight Ameliorated the cardia injury Reduced cardiac lipid accumulation, deposition of collagen, oxidative stress, and cardiac fibrosis Downregulated the proinflammatory cytokines	↑ T-SOD ↓ MDA ↓ TGF-*β*, collagen-1, fibronectin, NF-*κ*B, TNF-*α*, and IL-1*β*	[84]
In vivo	Male STZ-induced diabetic Kunming male mice	75, 150, and 300 mg/kg b.w.	Decreased the ratio of heart to body weight Ameliorated the cardia injury Reduced cardiac lipid accumulation, deposition of collagen, oxidative stress, and cardiac fibrosis Downregulated the proinflammatory cytokines	↑ T-SOD ↓ MDA ↓ TGF-*β*, collagen-1, fibronectin, NF-*κ*B, TNF-*α*, and IL-1*β*	[84]
In vivo	Male Sprague-Dawley rats with ACHSFD-induced metabolic hypertension	400 and 600 mg/kg b.w.	Lowered blood pressure Improved lipid abnormalities, intestinal flora, and the vascular endothelial relaxation function	↑ SCFA-GPCR43/41 pathway	[75]
In vivo	HFD/STZ-induced diabetic mice	75, 150, and 300 mg/kg b.w.	Reduced cardiac injury and fibrosis Suppressed insulin resistance Accelerated lipid transport	↑ PPAR-*α*, p-IRS1, and E-cadherin ↑ HDL-C ↓ TC, TG, and LDL-C ↓ TGF-*β*1, p-JNK, Twist, Snail1, and Vimentin	[86]
*Liver protection*					
In vivo	Male Kunming mice	50, 100, and 200 mg/kg b.w.	Mitigated the alcohol-induced liver injury Reduced the degeneration, inflammatory infiltration, and lipid droplet accumulation in liver	↑ GSH, SOD, GSH-Px, and CAT ↑ Nrf2, HO-1, and NQO1 ↓ ALT, AST, TC, and TG ↓ MDA ↓ CYP2E1 ↓ TLR-4 and NF-*κ*B p65	[89]
*Neuroprotection*					
In vivo	Male and female neonatal Sprague-Dawley rats	75, 150, and 300 mg/kg b.w.	Protected against hypoxic-ischemic brain damage Alleviated the impaired neurobehaviors and antioxidant capacity Inhibited the neuronal apoptosis Enhanced the expression of neurotrophic factors	↑ SOD ↑ Bcl-2 ↑ KCC2 ↓ NOS, NO, and MDA ↓ Cleaved caspase-3 and Bax ↓ HIF-1*α* and HDAC1	[96]
In vivo	Male ICR mice	1 and 3 g/kg b.w.	Reduced the depression-like behavior (decreased sucrose consumption and increased immobile time)	↑ NGF and BDNF	[98]
In vitro	PC12 cells	1, 3, and 10 *μ*g/mL	Potentiated the neurite outgrowth treatment	↑ Neurofilaments	
*Antifatigue*					
In vivo	Male BALB/c mice		Improved the fatigue resistance Increased the antioxidant activity Inhibited the decrease in glycogen storage	↑ PGC-1*α*	[103]

NA: not applicable.

**Table 2:** The health benefits and related molecular mechanisms of *Dendrobium officinale* polysaccharides.

Type of study	Object	Dosage	Effects	Potential mechanisms	References
*Anticancer*					
In vitro	Colon cancer cell line CT26 cells	0, 400, and 800 *μ*g/mL	Induced mitochondrial dysfunction and autophagy Reduced the cell proliferation	↑ ROS-AMPK-autophagy pathway	[57]
In vitro	Colon cancer cell line HT-29 cells	25, 50, 100, 200, and 400 *μ*g/mL	Inhibited the proliferation of cells Induced cell apoptosis	↑ Mitochondria-dependent intrinsic apoptotic pathway	[59]
In vivo	Zebrafish	27.8, 83.3, and 250 *μ*g/mL	Inhibited tumor metastasis	NA	
In vivo	Male BALB/c mice with AOM/DSS-induced colorectal cancer	50, 100, and 200 mg/kg b.w.	Alleviated chronic colitis and colon damage Reduced the formation and growth of colon tumor Restored the intestinal barrier function Improved antitumor immune response in the tumor microenvironments	↑ ZO-1 and occludin ↑ Metabolic ability of tumor infiltrated CD8(+) CTLs ↑ PD-1	[58]
In vivo	Male Wistar rats	2.4 and 4.8 g/kg b.w	Inhibited the gastric carcinogenesis Exerted the antioxidative effect Induced cell apoptosis	↑ GSH-Px and IL-2 ↑ IL-10 ↑ Bax and caspase-3 ↓ 8-OHdG and MDA ↓ Activin A, Agrin, IL-1*α*, ICAM-1, and TIMP-1 ↓ Bcl-2	[119]
In vitro	Liver hepatocellular carcinoma cell line HepG2 cells	50, 100, 200, and 400 *μ*g/mL	Inhibited cell growth Induced apoptosis Altered mitochondrial function	↑ ROS ↑ Bax ↓ Bcl-2	[53]
In vitro	Human osteosarcoma cell line U2OS and Saos-2 cells	12.5, 25, 50, 100, and 200 *μ*g/mL	Inhibited the proliferation of cells Increased cisplatin-induced cell apoptosis	↑ p53, Bax, and Bak ↑ The ratios of cleaved caspase-9 to caspase-9, cleaved caspase-3 to caspase-3, and cleaved PARP to PARP ↓ Bcl-2 and Mcl-1	[54]
In vitro	Human cervical carcinoma HeLa cells	25, 50, 100, 200, and 400 *μ*g/mL	Inhibited the proliferation of cells Induced the apoptosis	↑ ERK, JNK, and p38	[12]
In vivo	Male Wistar rats	2.4, 4.8, and 9.6 g/kg b.w.	Prevented MNNG-induced PLGC Reduced liver and kidney damage	↑ Nrf2 signaling pathway ↑ HO-1 and NQO-1 **↓** 8-OhdG	[34]
In vivo	Male Sprague-Dawley rats	2.4, 4.8, and 9.6 g/kg b.w.	Inhibited MNNG-induced PLGC Modulated serum endogenous metabolites	↓ Wnt2*β*, Gsk3*β*, PCNA, and CyclinD1	[55]
*Antidiabetes*					
In vivo	Streptozotocin-induced diabetic male Sprague-Dawley rats	25 and 100 mg/kg b.w.	Lowered the fasting blood sugar levels Increased serum insulin and GLP-1 secretion	Ca^2+^/CaM/CaMKII and MAPK signaling pathways	[69]
In vitro	Murine enteroendocrine cell line STC-1 cells	0, 0.2, 2, 20, 200, and 2000 *μ*g/mL			
In vivo	Streptozotocin-induced diabetic male Wistar rats	20, 40, 80, and 160 mg/kg b.w	Decreased the levels of fasting blood glucose, insulin, glycated serum protein, and serum lipid profile Alleviated the pancreatic injury Reduced the oxidative stress injury	↑ SOD ↓ MDA	[36]
In vivo	Male HFD/STZ-induced diabetic C57BL/6J mice	100, 200, and 400 mg/kg b.w.	Promoted hepatic glycogen synthesis Reduced the degradation of hepatic glycogen and hepatic gluconeogenesis Reversed the instability of the liver glycogen structure Ameliorated hepatic glucose metabolism	NA	[68]
*Gastrointestinal protection*					
In vitro	Human gastric mucosal epithelial HFE145 cells	31.25, 62.5, 125, 250, and 500 *μ*g/mL	Ameliorated H_2_O_2_-induced apoptosis Decreased the number of apoptotic cells in both early and late apoptosis stages Improved the nuclei morphology changes	↑ Bcl-2 **↓** ROS, caspase-3, PARP cleavage, and Bax **↓** NF-*κ*B activation	[33]
In vivo	Male Sprague-Dawley rats	124 and 248 mg/kg b.w.	Reduced the ethanol-induced gastric mucosal injury, mucin loss, and apoptosis	↓ The ratio of Bax to Bcl2	
In vivo	Female ICR mice	0.5 and 2 mg/kg b.w.	Regulated the small intestinal immune function Modulated intestinal mucosal structures Influenced the production of immune cytokine production	NA	[16]
In vivo	Male BALB/c mice	200 mg/kg b.w.	Improved the diversity of gut microbiota Alleviated dextran sulfate sodium-induced colitis	↑ SCFAs ↑ GPRs ↑ *Bacteroides*, *Lactobacillus*, and *Ruminococcaceae* cTNF-*α*, IL-6, IL-1*β* ↓ *Proteobacteria*	[74]
In vivo	Male Sprague-Dawley rats	100 and 400 mg/kg b.w.	Reduced gastric mucosal injury score and pathological injury Increased the antioxidant activity	↑ p-AMPK, LC3*β*, HO-1, and Beclin-1 ↑ Bcl-2 ↓ p-mTOR and p62 ↓ Caspase-3 and Bax	[80]
In vitro	Human gastric epithelial cell line GES-1 cells	62.5, 125, and 250 *μ*g/mL	Alleviated cell apoptosis		
*Cardiovascular protection*					
In vitro	H9c2 cardiomyocytes	6.25, 12.5, and 25 *μ*g/mL	Increased the survival rate of cells and antioxidant enzyme activity Reduced the LDH leakage, lipid peroxidation damage, ROS production, and the mitochondrial membrane potential Ameliorated H_2_O_2_-induced apoptosis	↑ The ratios of p-Akt to Akt and p-ERK to ERK ↑ The ratios of Bcl-2 to Bax ↓ The ratios of p-p38 to p38, p-JNK to JNK, and p-PI3K to PI3K	[82]
*Liver protection*					
In vivo	Male Wistar rats	20, 40, 80, and 160 mg/kg b.w.	Ameliorated the liver metabolism Balanced the metabolism of ceramide and bile acids Reduced oxidative stress, inflammation, and hepatic lipid accumulation	NA	[85]
In vivo	Male ICR mice	50, 100, and 200 mg/kg b.w.	Attenuated acetaminophen-induced liver injury Triggered the dissociation of Nrf2 from Nrf2-Keap1 complex Promoted the nuclear translocation of Nrf2	↑ GSH and CAT ↑ GCLC, GCLM, HO-1, and NQO1 ↑ Nrf2-Keap1 signaling pathway ↓ ALT, AST, ROS, MDA, and MPO	[87]
In vivo	Male C57BL/6J	100, 200, and 400 mg/kg b.w.	Maintained the balance of GSH content in liver Protected against acute alcoholic liver injury	NA	[88]
In vivo	Male ICR mice	0.6 g/kg b.w.	Decreased TG and FA content in the liver Reduced C16:0/C14:0 and C18:1/C18:0 in FAs Increased C20:4/C20:3 and C22:4/C22:3 in FAs Inhibited the saturated FAs Improved the dysregulated levels of major phospholipids in the liver	↑ CPT1-*α* and ACOX1 ↓ FAS, SCD-1, and FATP2	[90]
*Lung protection*					
In vivo	Male Sprague-Dawley rats	200 mg/kg b.w.	Alleviated bleomycin-induced pulmonary inflammation and fibrosis Reduced the transformation of rat alveolar epithelial type II cells into myofibroblasts	↓ TGF-*β*1-Smad2/3 signaling pathway ↓ Smad2/3, p-Smad2/3, collagen I, and fibronectin	[94]
In vitro	Human bronchial epithelial cells	0.01, 0.1, and 1 *μ*g/mL	Ameliorated the cigarette smoke-induced mucus hypersecretion and viscosity	↓ MUC5AC ↓ Mucus secretory granules	[91]
In vivo	Male Sprague-Dawley rats	50 and 200 mg/kg b.w.			
In vitro	Mouse lung epithelial cells	0.01, 0.1, and 1 *μ*g/mL	Ameliorated the lung functions and inflammation in chronic obstructive pulmonary disease	↑ AQP5 ↓ MUC5AC	[92]
In vivo	Sprague-Dawley rats	5, 10, and 20 mg/mL			
A randomized, double-blind, and placebo-controlled clinical trial	Active cigarette smokers over 40 years old with mild airflow obstruction	1.2 g thrice daily			
*Neuroprotection*					
In vivo	Pentetrazol-induced epileptic male Sprague-Dawley rats	1.5 g/kg b.w.	Reduced brain inflammation and seizures	↓ IL-1 and TNF-*α* ↓ MAPK signaling pathways	[41]
In vivo	Female Kunming mice	140 mg/kg b.w.	Ameliorated the learning and memory disability Reduced the oxidative stress and neuroinflammation	↑ Nrf2/HO-1 pathway	[97]
*Antiosteoporosis*					
In vitro	Bone marrow mesenchymal stem cells	100, 200, and 400 *μ*g/mL	Enhanced osteogenic differentiation of BMSCs Inhibited adipogenic differentiation	↑ Nrf2 signaling pathway	[100]
In vivo	Fifteen-month-old mice	150 mg/kg b.w.	Increased the bone mass Reduced the accumulation of marrow adipose tissue and oxidative stress Prevented the age-related osteoporosis		
In vitro	RAW264.7 cells	40 and 80 *μ*g/mL	Alleviated estradiol deficiency Maintained calcium and phosphorus homeostasis Improved uterine and femoral physical parameters and bone microarchitecture Inhibited osteoclastogenesis and the expression of some osteoclast-specific genes	NA	[101]
In vivo	Female Wistar rats	150, 300, and 600 mg/kg b.w.			
*Antiobesity*					
In vitro	Hepatocytes, C2C12 myoblasts, and 3T3-L1 preadipocytes	200 *μ*g/mL	Ameliorated the insulin resistance	↑ PPAR-*γ* ↑ HDL-C ↓ TG, FFA, TC, and LDL-C	[104]
In vivo	Male C57BL/6 mice and ob/ob mice	150 mg/kg b.w.	Reduced insulin resistance and visceral adipose tissue inflammation Decreased the HFD-induced liver lipid accumulation		
*Laxation*					
In vivo	Male and female ICR mice	29, 57, and 114 mg/kg b.w.	Attenuated constipation Increased the gastrointestinal transit ratio Improved the fecal output characteristics	↑ Motilin, gastrin, acetyl cholinesterase, and substance P ↓ Somatostatin	[105]

NA: not applicable.

## Abbreviations

8-OHdG: 8-Hydroxy-deoxyguanosine
ACHSFD: Overconsumption of alcohol and high sugar and fat diets
ACOX1: Aryl-coenzyme A oxidase
ALT: Alanine aminotransferase
AMPK: AMP-activated protein kinase
AQP5: Aquaporin 5
AST: Aspartate aminotransferase
Bak: Bcl-2 homologous antagonist killer
Bax: Bcl-2-associated X protein
Bcl-2: B-cell lymphoma-2
Bcl-xL: Bcl-extra large
BDNF: Brain-derived neurotrophic factor
Bik: Bcl-2 interacting killer
BMSCs: Bone marrow mesenchymal stem cells
BUN: Blood urea nitrogen
CaM: Calmodulin
CaMK: Calmodulin-dependent protein kinase
CAT: Catalase
CCL4: Chemokine (C-C motif) ligands 4
CK: Creatine kinase
CPT1-*α*: Carnitine palmitoyltransferase 1-alpha
CTLs: Cytotoxic T lymphocytes
DSS: Dextran sulfate sodium
ERK: Extracellular signal-regulated kinases
FAS: Fatty acid synthase
FATP2: Fatty acid transport protein 2
FFA: Free fatty acid
GCLC: Glutamate-cysteine ligase catalytic subunit
GCLM: Glutamate-cysteine ligase regulatory subunit
GLP-1: Glucagon-like peptide-1
GPCR: G-protein-coupled receptor
GSH: Glutathione
GSH-Px: Glutathione peroxidase
Gsk3*β*: Glycogen synthase kinase 3 beta
HDAC1: Histone deacetylase 1
HDL-C: High-density lipoprotein cholesterol
HFD: High-fat diet
HIBD: Hypoxic-ischemic brain damage
HO-1: Heme oxygenase 1
ICAM-1: Intercellular adhesion molecule 1
IFN-*α*: Interferon alpha
IgM: Immunoglobulin M
IL-1*β*: Interleukin-1 beta
IP-10: Interferon gamma-induced protein 10
JNK: Jun N-terminal kinase
KCC2: K^+^-Cl^−^ cotransporter 2
LD: Lactic acid
LDH: Lactate dehydrogenase
LDL-C: Low-density lipoprotein cholesterol
MAPK: Mitogen-activated protein kinases
MDA: Malonaldehyde
MNNG: 1-Methyl-3-nitro-1-nitrosoguanidine
MPO: Myeloperoxidase
MUC5AC: Mucin-5AC
MyD88: Myeloid differentiation factor
NAFLD: Nonalcoholic fatty liver diseases
NF-*κ*B: Nuclear factor kappa-B
NGF: Nerve growth factor
NO: Nitric oxide
NOS: Nitric oxide synthase
NQO-1: NAD(P)H: quinone oxidoreductase-1
Nrf2: Nuclear factor erythroid 2-related factor 2
PARP: Poly (ADP-ribose) polymerase
PCNA: Proliferating cell nuclear antigen
PD-1: Programmed death-1
PGC-1*α*: Peroxisome proliferator-activated receptor gamma coactivator 1-alpha
PI3K: Phosphoinositide 3-kinases
PLGC: Precancerous lesions of gastric cancer
p-IRS1: Phosphorylation of insulin receptor substrate 1
ROS: Reactive oxygen species
SCD-1: Stearoyl-coenzyme A desaturase-1
SCFA: Short-chain fatty acid
TC: Total cholesterol
TG: Triglyceride
TGF-*β*1: Transforming growth factor beta 1
TIMP-1: Tissue inhibitor of matrix metalloproteinase-1
TLR4: Toll-like receptor 4
TNF-*α*: Tumor necrosis factor-alpha
TRAF-6: Tumor necrosis factor receptor-associated factor-6
ZO-1: Zonula occludens-1.
